# Extraction of Nucleotides from Dietary Supplements by Newly Synthesized Adsorbents

**DOI:** 10.3390/foods12193675

**Published:** 2023-10-06

**Authors:** Sylwia Studzińska, Szymon Bocian, Paulina Stypczyńska, Andrzej Wolan

**Affiliations:** 1 Chair of Environmental Chemistry and Bioanalytics, Faculty of Chemistry, Nicolaus Copernicus University in Toruń, 7 Gagarin Str., PL-87-100 Toruń, Poland; bocian@chem.umk.pl (S.B.);; 2 Chair of Organic Chemistry, Faculty of Chemistry, Nicolaus Copernicus University in Toruń, 7 Gagarin Str., PL-87-100 Toruń, Poland; wolan@chem.umk.pl

**Keywords:** nucleotides, dispersive solid-phase extraction, new adsorbents, electrostatic interactions, dietary supplements

## Abstract

The aim of the study was the synthesis and application of novel adsorbents for the extraction of nucleotides from dietary supplements. Three different adsorbents modified with a silane containing two amine groups and various dicarboxylic acids were synthesized and characterized using various instrumental techniques. Next, different solvents were tested for their adsorption and desorption of five nucleotides. The results showed that the efficiency of both processes depends on the conditions used and the type of dicarboxylic acid bound to the surface of the adsorbent. The best results were obtained for succinic acid. The most effective adsorption occurred for water acidified with acetic acid to pH 4.5, while the highest recoveries (85–102%) with high reproducibility were obtained for 10 mM ammonium acetate at pH 9. The nucleotide extraction was performed simply by changing the charge at the adsorbent surface, providing the possibility of electrostatic attraction and repulsion between the adsorbent and nucleotides. Moreover, the sorption capacity of the obtained materials was also determined, which was essential for their use in extracting nucleotides from real samples by dispersive extrusion to the solid phase. The new adsorbents and the developed extraction method were successfully applied to isolate nucleotides from two different dietary supplements with different compositions (one of them with yeast strains). The method is simple and reproducible; no organic solvents or high-concentration inorganic salts are used (it is environmentally friendly). The entire process is performed in one centrifuge tube and is cheaper compared with methods used so far.

## 1. Introduction

Nucleotides are esters of nucleosides and phosphoric acid [1]. They play an essential role in synthesizing proteins and phospholipids and in the transformation of sugars, and they are also involved in regulating metabolism [2,3,4]. In addition, studies have shown that these compounds are essential for supporting immune system function, the regeneration and function of the small intestine, and supporting healthy wound healing [3,5,6,7]. Nucleotides also induce and protect the hematopoietic system, support platelet aggregation, and have strong anti-inflammatory and antiviral properties [1,5]. Due to their biological activity, they have found an application as an ingredient of modified milk for infants [3,6,7]. In the pharmaceutical industry, they are found as dietary nucleotides in supplements, with the most common being 5’-monophosphate uridine (UMP) and cytidine 5’-monophosphate (CMP). Supplementation with these preparations is recommended when the body needs rapid restoration [8]. 

The use of nucleotides in dietary supplements or food has led to the development of new methods for their analysis, especially regarding sample preparation. In general, protein precipitation or the removal of lipids by liquid–liquid extraction (hexane, chloroform) is needed in some cases as one of the first steps of sample purification [9,10,11]. However, the mainly used method for nucleotide extraction is solid-phase extraction, where adsorption plays a key role [12]. Both efficient purification and enrichment depend on the affinity of the analytes for the adsorbents, e.g., silica- or cellulose-based materials [12,13,14,15,16,17]. Therefore, the development of new materials as adsorbents has a significant impact on the solid-phase extraction (SPE) and dispersive solid-phase extraction (dSPE) techniques, not only to improve efficiency and selectivity but also to increase chemical or physical stability [12].

An anion-exchange SPE is most commonly used to purify nucleotides in biological matrices and to supplement formulations. Weak anion exchangers (e.g., amine) or strong ones (e.g., quaternary ammonium salts) were developed for this purpose. Still, they often require high concentrations of inorganic salts for nucleotide elution, such as 300 mM NH_4_H_2_PO_4_ or ammonium hydroxide at high pH [13,14]. This often results in the need for an additional step involving desalting before the final analysis by, e.g., mass spectrometry. On the other hand, they have proved to be suitable materials for the simultaneous extraction of mono-, di-, and triphosphates [14]. The application of polymeric adsorbents, e.g., Strata X (with hydrophilic and hydrophobic groups), may overcome the need to use high concentrations of inorganic salts for nucleotide extraction [18]. The ion-pair mode is commonly applied for these (or other non-polar) materials with solutions of amines (usually ethanolamine, 25–100 mM, pH 6–8) and methanol in the eluents [18,19]. Ion-exchange and ion-pair modes for nucleotide extraction showed to be effective for biological matrices (human cerebrospinal fluid) and other matrices [14,18]. The recoveries are high (above 90%), and the removal of matrix interferences is fair [14,18,19]. Another example is the application of Oasis^®^ WAX (a polymeric adsorbent with anion-exchange ligands), which allows the retention and release of strongly acidic compounds. Elution of nucleotides could be carried out with a mixture of 0.25% ammonia solution (pH 10) and acetonitrile. The recovery ranged from 70% to 105% [20]. 

Apart from SPE, dSPE has also been used for nucleotide extraction, providing essential advantages over SPE: the small amounts of sorbent and solvent required, which reduce handling and costs. Charcoal has been applied for the adsorption of nucleotides from various matrices in dSPE [21,22]. This process is based on the π–π interaction of the aromatic ring of nucleobases and the structure of charcoal. However, the effective elution of these compounds requires 2% NH_3_ and 50% acetonitrile in water. Although no highly concentrated inorganic salts are needed, the high volume of organic solvent is indispensable [22].

SPE has been successfully used to extract nucleotides; however, research on new adsorbents for isolating nucleotides is essential and needed. For this reason, the study’s goal was to synthesize novel adsorbents with amino and carboxyl groups on the surface, which could be applied to the dSPE of nucleotides. The idea behind the synthesis was to extract nucleotides by changing the charge at the adsorbent surface, providing the possibility of electrostatic attraction and repulsion between the adsorbent and nucleotides. Both the designed adsorbent and the research idea are new in nucleotide extraction attempts. The adsorbent was synthesized for the first time and its application to the isolation of studied compounds is also the novelty of the present research. Moreover, the application of the synthesized adsorbents to the extraction of studied compounds from dietary supplements was also attempted.

## 2. Materials and Methods

### 2.1. Reagents

Nucleotide standards adenosine-5′-monophosphate (AmP), cytidine-5′-monophosphate (CmP), uridine-5′-monophosphate (UmP), guanosine-5′-monophosphate (GmP), and inosine-5′-monophosphate (ImP) were purchased from Sigma-Aldrich (Dorset, UK). Stock solutions (500 µg/mL) of nucleotides were prepared by dissolving them in water to a 50 µg/mL concentration. 

Mobile phases used during the study were prepared using methanol (LC-gradient-grade purity), ammonium acetate, and acetic acid (both of HPLC purity) (Sigma-Aldrich, Dorset, UK). Deionized water was obtained from the Milli-Q system (Millipore, El Passo, TX, USA). The following reagents were used for sample preparation: ammonium acetate (95% purity), 25% solution of ammonia (95% purity), 50% solution of acetic acid (95% purity), methanol, ethanol, and acetonitrile (for analysis) (Sigma-Aldrich, Dorset, UK).

Reagents used for the adsorbent synthesis were as follows: hydrochloric acid (HCl) (for MES pH correction) (Stanlab, Lublin, Poland), sodium hydroxide (Avantor, Gliwice, Poland), (1-ethyl-3-(3-dimethylaminopropyl)carbodiimide (EDC) (Merck, Darmstadt, Germany), morpholineethanesulfonic acid (MES), 3-(2-aminoethylamino)propyl]-trimethoxysilane, malonic acid, succinic acid, and glutaric acid (Sigma-Aldrich, St. Louis, MO, USA). The synthesis was performed on a silica gel with particle diameter 63–200 µm, pore diameter 60 Å, pore volume 0.7–0.85 mL/g, and an average surface area around 500 m^2^/g (Sigma-Aldrich, St. Louis, MO, USA).

### 2.2. Instrumentation

The UltiMate^®^ 3000 Binary Rapid Separation LC (RSLC) (Dionex, Sunnyvale, CA, USA) UHPLC system, including a binary pump and a DAD-3000RS Diode Array Detector was used during the study. Chromatographic data were collected with the use of the Chromeleon 7 program. Moreover, the ultra-high-performance liquid chromatograph Shimadzu Nexera UHPLC (Kyoto, Japan) equipped with two pumps (LC-30AD), degasser (DGU-20A5R), autosampler (SIL-30AC), column thermostat (CTO-30A), and spectrophotometric diode-array UV-Vis detector (SPD-M20A) was used for the analysis of supplement extracts. The data were collected using LabSolution version 5.8 with a computer data acquisition station. Sample centrifugation was performed using the 5424 microliter Eppendorf AG centrifuge (Hamburg, Germany) and CentriVap vacuum concentrator (Labconco, Kansas City, MO, USA). The laboratory CP-505 pH meter (Elmetron, Zabrze, Poland) and shaker IKA^®^ Vortex Genius 3 (IKA Works GmbH & Co., Warsaw, Poland) were also used in the study.

Elementary analysis was performed using Vario MACRO CHN (Elementar Analysensysteme GmbH, Langenselbold, Germany). FTIR spectra were registered on FT-IR Vertex 70V with a Hyperion 1000/2000 microscope in the range 4000–400 cm^−1^ (Bruker Optics GmbH & Co. KG, Leipzig, Germany). Spectra were registered in transmission mode using a potassium bromide pellet. ^13^C solid-state CP MAS NMR spectra were recorded on a Bruker Avance III 700 MHz spectrometer (Bruker BioSpin, Billerica, MA, USA).

The greenness of the developed method was determined according to the AGREEprep (Analytical Greenness Metric for Sample Preparation) software (Gdańsk University of Technology, Gdańsk, Poland), available online free of charge [23].

### 2.3. Chromatographic Conditions

The reverse-phase ultra-high-performance liquid chromatography (RP UHPLC) was used to determine nucleotides. The octadecyl stationary phase with a terminal pentafluorophenyl group ACE Excel C18PFP (1.7 µm, 100 × 2.1 mm) (Advanced Chromatography Technologies, Aberdeen, UK) was applied, and studies were carried out with a mobile phase containing methanol (MeOH) and 50 mM ammonium acetate of pH 5.0 or 5.5. Gradient elution was used by increasing the methanol content 0–10% *v*/*v* MeOH for 10 min. The mobile phase flow rate was 0.3 mL/min. The injection volume was 1 µL. The temperature of the autosampler was 10 °C, while the column thermostat temperature was 30 °C. The UV-Vis detection wavelength was selected as λ = 254 nm. 

### 2.4. Synthesis of New Adsorbents

**Figure 1:**
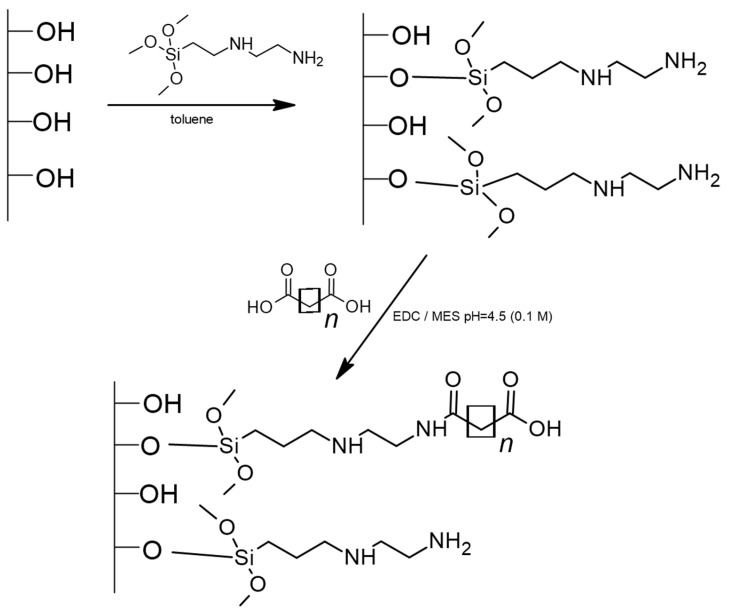
The schematic synthetic route of new adsorbents.

The adsorbent synthesis was carried out in two steps. In the first step, silica gel was modified with a silane containing two amine groups. This is a well-known procedure that has been described elsewhere [24,25]. In the second step, amine groups were reacted with dicarboxylic acids (in a 2:1 proportion of primary amines to carboxylic acid). In detail, the surface of bare silica was chemically modified with 3-(2-aminoethylamino)propyl]trimethoxysilane under vacuum in a glass reactor, which provides complete insulation from the external environment. Synthesis conditions were set according to the earlier methodology [24,25]. The intermediate products were washed with toluene, methanol, and hexane through a Schott funnel to eliminate the excess reactants. In the second step, the prepared support was bonded with dicarboxylic acid in the presence of EDC in a solution of 0.1 M MES (pH = 4.5) in a glass flask heated to a temperature of 40 °C. The reaction was carried out for 24 h. The product was washed first with water, then twice with the solution of 0.1 M MES (pH = 4.5) and once again with water to eliminate the excess reactants. Finally, the adsorbent was dried in a laboratory dryer at 60 °C. Three different dicarboxylic acids—malonic acid C3COOH, succinic acid C4COOH, and glutaric acid C5COOH—were bonded to the silica surface, obtaining three different adsorbents. Figure 1 shows the schematic route of the synthesis process.

### 2.5. Nucleoside Adsorption and Desorption Tests for New Adsorbents 

The experiment for the determination of time needed to reach adsorption equilibrium (adsorption kinetics) was carried out in similar conditions to the first step of the extraction procedure. Aliquots of 500 µL of 50 µg/mL AmP or GmP standards were added to 10 mg of adsorbent (C4COOH) and mixed for 40 s. Next, 50 µL of the solution above the adsorbent were taken at given time intervals (2, 4, 6, 8, 10, 20, and 30 min) and centrifuged for 3 min at 10,000× *g*.

Adsorption of different nucleotides at the surface of C3COOH, C4COOH, C5COOH was performed in various solvents. An amount of 200 µL of the 50 µg/mL tested compound was added to 5 mg of adsorbent, mixed by vortexing for 40 s, and centrifuged for 10 min at 10,000× *g*. Next, the supernatant was analyzed by RP UHPLC to determine how much nucleotide was adsorbed by the adsorbent. The adsorption process conditions were optimized for AmP and GmP, and the impact of salt type and pH was tested. Each experiment was performed at least twice.

After adsorption, the adsorbent was washed with 200 µL of 10 mM ammonium acetate (pH 4.5) to remove non-specifically bound compounds. The suspension was centrifuged at 10,000× *g* for 10 min, and the supernatant was removed and analyzed by RP UHPLC.

The nucleotide desorption from the newly synthesized adsorbents was carried out by adding 200 µL of solvent, mixing by vortexing for 1 min, and centrifuging for 10 min at 10,000× *g*. The influence of salt type and pH on nucleotide recovery was tested. Each experiment was performed twice. The concentration of nucleotides desorbed from the adsorbent was determined using RP UHPLC.

### 2.6. Adsorption Capacity

The adsorption capacity tests were carried out under optimized experimental conditions. These were determined for two different amounts of adsorbent: 1 or 2 mg. Aliquots of 100 µL of different concentrations of AmP (from 5 µg/mL to 80 µg/mL) were added to a given adsorbent portion. The tests were performed using three weighed portions. The value of the adsorption capacity Q_e_, [µmol/mg] in equilibrium was calculated by the following equation:

$$Q_{e}=\frac{(C_{0}-C_{e})\cdot V}{m}$$

where C_0_ is the initial AmP concentration [µmol/L], C_e_ is the equilibrium AmP concentration [µmol/L], V is the initial volume of sample solution [L], and m is the mass of the adsorbent [mg].

### 2.7. Nucleotides Extraction from Two Dietary Supplements

Two different dietary supplements were used during the study. S1 comes in the form of capsules comprising the yeast strain Saccharomyces boulardii, nucleotides, and insulin, while S2 comes in tablet form, which includes a nucleotide (UmP), B vitamins (B1, B12, B6), and folic acid. 

In the case of S1, one capsule was opened, and the powder was transferred quantitatively to a 100 mL volumetric flask and topped up with distilled water. Next, the flask was placed in an ultrasonic bath for 5 min. A quantity of 50.0 ± 0.1 mg of the C4COOH adsorbent was mixed with 100 µL of the sample solution and 400 µL of H_2_O (pH 4.5). The suspension was shaken for 1 min and centrifuged for 10 min. The supernatant was removed, and the adsorbent was washed with 500 µL of H_2_O (pH 4.5), shaken, and centrifuged. Next, 500 µL of 10 mM CH_3_COONH_4_ (pH 9.0) was added, and the suspension was mixed and centrifuged. Finally, the supernatant was analyzed by RP UHPLC.

In the case of S2, one tablet was crushed in a mortar and transferred quantitatively to a 100 mL volumetric flask, then topped up with distilled water. Next, the flask was placed in an ultrasonic bath for 5 min. A quantity of 50.0 ± 0.1 mg of the C4COOH adsorbent was mixed with 150 µL of the sample solution and 350 µL of H_2_O (pH 4.5). The sample was handled analogously to S1 in the next steps of the sample preparation procedure.

### 2.8. Recovery of Nucleotides from Dietary Supplements and Their Quantification

The quantification of nucleotides in the supplements was performed by the standard addition method. Known amounts of the standards were added to the samples in the range of about 50% to 150% of the expected content of the analytes to be determined. Six different concentrations were used to generate calibration curves in the range of 2.5–45.0 µg/mL. Linearity was estimated by the calculation of the determination coefficient (R^2^) for the calibration curve. The limit of quantification (LOQ) was determined as a signal approximately 10 times the noise level. 

The recovery of nucleotides using the developed dSPE method was determined and calculated based on comparing the results obtained for enriched extracts (fortified with nucleotides after extraction) and extracts from enriched dietary supplements (5 µg/mL). Next, the samples were analyzed by the chromatographic method developed during the study. Recovery was calculated from the following formula:

$$R=\frac{A_{1}-A_{3}}{{(A}_{2}-A_{3})}\times 100\%$$

where: R—recovery [%];

A_1_—peak area in the extract of dietary supplement enriched after the extraction;
A_3_—peak area in the dietary supplement extract;
A_2_—peak area in the dietary supplement extract enriched before the extraction.

## 3. Results and Discussion

### 3.1. Characterization of Adsorbents

The synthesized adsorbents were characterized by elemental analysis. Table 1 shows the results of chemical modification of the silica gel surface, i.e., carbon, nitrogen, and hydrogen content, after each bonding reaction. The resulting data allows for the calculation of the bonded ligand coverage density based on the equation derived by Berendsen [26]. The data presented in Table 1 confirm the bonding of individual carboxylic acids due to the increased carbon content in the synthesized stationary phases. Nitrogen is added only in the first step of the modification; thus, the nitrogen content is constant. The data also confirm the successful silica modification with silane. Based on the coverage density data, statistically, less than 50% of amine ligands reacted with the dicarboxylic acid. This is less than the assumed degree of reaction but allows a material to be obtained that possesses both amine and carboxylic groups on the surface. 

To verify the structure of the synthesized material, the FT IR spectra were obtained in transmission mode (pellet with potassium bromide). A comparison of the spectra is presented in Figure S1. A few characteristic signals may be observed. The signal at ṽ ≈ 3200–3600 cm^−1^ (maximum at 3427 cm^−1^) corresponds to stretching vibrations of the –OH group. This group is present in free carboxylic groups of bonded acids but also in the water that may have been adsorbed on the adsorbent. An additional band corresponding to the amine group may be observed at 1626 cm^−1^ (bending vibrations). The signal of the –NH group is also observed in this range. The signals at ṽ ≈ 1564 and 1708 cm^−1^ correspond to carbonyl-group stretching vibrations in the amide, confirming carboxylic acid’s bonding with amines. Unreacted methoxy groups in the Di-amine sample provide a signal in the range of 2582–2922 cm^−1^. These groups hydrolyze during further modifications; thus, the signals are strongly reduced. 

Transmission FT IR is not a method that guarantees identification, so the structures of the synthesized adsorbed materials were also confirmed using 13C CP MAS NMR spectra. Obtained spectra are presented in Figure S2. The quality of the spectra is low because the experiment was performed in the solid state. Obtaining a sharp signal for 13C in the solid state is impossible, even on the 700 MHz apparatus. Silica modified with 3-(2-aminoethylamino)propyl]-trimethoxysilane (Figure S2A) provides four main signals and one additional signal from an unreacted methoxy group at around 50 ppm. The methylene group bonded with the silicon atom produces a signal at 10 pmm, and the second carbon atom produces a signal at 25 ppm. Methylene groups bonded with nitrogen provide a signal in the range of 40–50 ppm. 

After the reaction with acids, additional signals are observed. The peak corresponding to the carbonyl group is the most important, which may be observed at around 180 ppm (Figure S2B–D). The carbonyl group provides a weak signal at 13C NMR because the carbon atom is not bonded with a hydrogen atom. Methylene groups in the acid give a signal in the range of 25–35 ppm [27,28]. 

**Figure 2:**
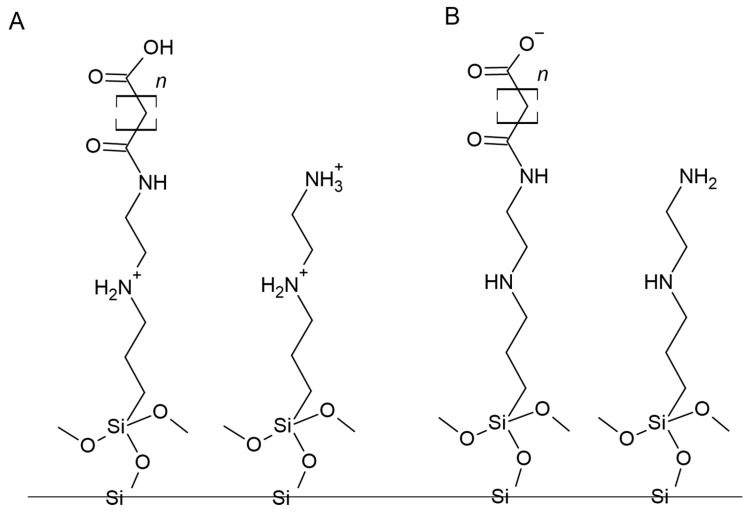
Structure of synthesized adsorbents in various pHs: during adsorption (acidic conditions) (**A**) and during desorption (alkaline conditions) (**B**). Notation: *n*—number of carbon atoms, equal to 1–3.

Based on the elemental analyses, FT IR spectroscopy, and 13C NMR spectroscopy, the structure of the obtained adsorbent was proposed. The resolution of instrumental techniques cannot recognize which amine groups were modified. Based on the information that EDC catalyzes the reaction preferentially with primary amines, the possible structure is illustrated in Figure 2.

### 3.2. Nucleotides Analysis

Nucleotides and nucleosides were analyzed using RP UHPLC. This method is based on a method previously developed by our team [11]. A stationary phase with octadecyl ligands with terminal pentafluorophenyl groups was applied. This provides for the retention of all analyzed compounds, both nucleotide anions and neutral nucleosides. This phenomenon is probably a consequence of multiple retention mechanisms with hydrophobic, π–π, and dipole–dipole interactions and hydrogen bonding. Since the development of a chromatographic method was not the purpose of this paper, anyone interested in the effects of different parameters on the retention of the two groups of compounds is welcome to read Ref. [11].

During the study of nucleotide adsorption and desorption from the surface of new adsorbents, solutions of single compounds were used, and the separation of their mixture was not crucial at that time. Figure S3A shows overlaid chromatograms of standard solutions of the individual nucleotides analyzed individually using the same chromatographic method. The limit of quantitation (LOQ) and limit of detection (LOD) were determined for the applied chromatographic method. LOD was appointed as the concentration of analyte that yields an analytical signal value three times higher than the noise level, while LOQ is the concentration yielding signals 10 times higher than noise. The LOD for nucleosides was in the range of 1.05–2.2 µg/mL, while the LOQ was 3.46–7.26 µg/mL. In the case of nucleotides, the LOD was within the range of 1.0–3.2 µg/mL, and the LOQ was between 3.3–10.6 µg/mL. However, to apply the developed extraction method to the dietary supplements, this method was modified to separate four nucleotides (CmP, UmP, GmP, and AmP). The gradient elution program and pH of the mobile phase were optimized. Figure S3B presents the separation of these compounds applied during the study. 

### 3.3. Development of a dSPE Method for Nucleotides Using New Adsorbents

#### 3.3.1. Adsorbent Structure and Probable Impact on Nucleotide Extraction

The structure of the surface groups located on the surface of the adsorbents was designed so that there were two groups, each with a positive or negative charge depending on the pH of the applied solvent. This was designed to increase the adsorption and desorption effects. Consequently, residual amines with pKa ≈ 9 and carboxyl with pKa ≈ 5 were selected and immobilized at the surface after the adsorbent synthesis. Since nucleotides are anions (above pH 3.8, since the pKa equals about 3.8), we aimed to retain them at the surface based on electrostatic attraction and release them based on electrostatic repulsion. Based on pKa values, it may be concluded that in solvents with pH lower than 9, –NH_2_ groups will be protonated, while at pHs greater than 5, –COOH groups will be ionized. Therefore, these adsorbents should be weak anion exchangers with a specific pH range (between 5 and 9), where they should have zwitterionic properties. Thus, at a pH between 4 and 5, the surface is positively charged (protonated amines) and attracts nucleotides that are still ionized and that prompt the adsorption. The increase in the pH results in the loss of the positive charge from the amines and the ionization of the carboxylic groups. The loss of positive charges and the presence of negative ones cause the repulsion of oligonucleotides from the adsorbent surface.

#### 3.3.2. Selection of Solvent Additive for Adsorption

Initial studies were conducted for two nucleotides: GmP and AmP. The stock solutions of these compounds were diluted 1:10 with the appropriate solvent to a final concentration of 50 µg/mL. The adsorption was carried out for 10 min, as this was the time designated as necessary to reach equilibrium (Figure S4). Due to the considerations connected with the structure of the adsorbent and the anionic character of the nucleotides, the following solvents with pH lower than 6 were tested: H_2_O pH = 4.5, 10 mM CH_3_COONH_4_ pH 4.5 and 10 mM CH_3_COONH_4_ pH 5.5. The percentage of nucleotide adsorbed on the material surface was determined based on the chromatographic results for the standards and samples (at similar concentrations) after the adsorption tests. The results are presented in Table 2.

**Figure 3:**
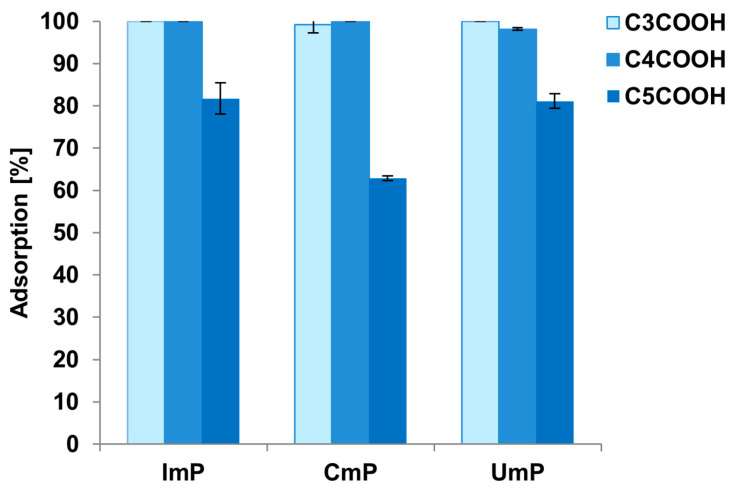
Summary of ImP, CmP, and UmP adsorption results from water at pH = 4.5 for all synthesized adsorbents.

Nucleotides were adsorbed at the surface of each adsorbent in the case of each applied condition, but this adsorption was not always complete. The lowest adsorption was observed for 10 mM CH_3_COONH_4_ pH = 5.5, while the highest was for water acidified to pH 4.5 (acidified with acetic acid) (Table 2). This effect was similar for all of the adsorbents, independently of the type of dicarboxylic acid bonded in the second step of the synthesis. The lower adsorption at pH 5.5 may be a consequence of the gradual dissociation of carboxyl groups, which reduces the strength of electrostatic interactions between the protonated amino groups and deprotonated phosphate groups in nucleotides. Finally, water acidified to pH = 4.5 was selected as a solvent for the adsorption of nucleotides. However, it must be underlined that 10 mM CH_3_COONH_4_ pH = 4.5 could also have been successfully used since it seems that, per our expectations, the proper acidic pH is the crucial parameter for nucleotide adsorption at the surface. This result is probably related to the positive charge of amino groups and their electrostatic attraction to negatively charged analytes. Figure 3 presents the adsorption results of three other nucleotides (UmP, CmP, ImP) for each adsorbent. 

These results show that adsorption on newly synthesized adsorbents is controlled not only by the pH of the solution but also by the type of dicarboxylic acids bonded to the surface in the second stage of synthesis (Figure 3). The lowest values were obtained for the C5COOH adsorbent, indicating that it is unsuitable for nucleotide extraction due to its limited adsorption. This material has the highest coverage density, and after the second synthesis stage, there is more carbon on its surface than on the other two adsorbents (Table 1). The higher carbon content results from the larger number of carbon atoms in the acid structure. On the other hand, the amount of residual amino groups on the surface of C5COOH (after the first stage of synthesis) is probably smaller than that for C3COOH and C4COOH. This effect could be responsible for the smaller adsorption of nucleotides based on electrostatic attraction.

The adsorption capacity was also measured for the tested adsorbents and selected nucleotide (AmP). The highest value was determined for the C5COOH—1.818 µg/mg. For C3COOH and C4COOH, these values were 0.793 and 0.989 µg/mg, respectively. In addition, the adsorbents were washed with 200 µL of H_2_O (pH 4.5) after the adsorption step, and no desorption was observed, proving that this solvent may be successfully used for washing during extraction.

#### 3.3.3. Selection of Solvent for Desorption

Regarding the desorption of nucleotides, only the solvents of basic pH were considered. According to our assumptions, we expected nucleotide desorption to be possible by changing the pH of the elution solution. We aimed for the amine and carboxyl groups to be deprotonated on the surface of the adsorbent. Deprotonation would lead to electrostatic repulsion of the nucleotides and their release into the solution. The pKa of the amine group is about 9; therefore, the following solutions for the desorption tests were selected: alkalized water (pH 10.0 and 10.5) and a 10 mM solution of CH_3_COONH_4_ with pH 9.0, 9.5, 10.0, or 10.5. All these solutions were alkalized with 10% of ammonia hydroxide. Initial tests were performed for two nucleotides: GmP and AmP. Each experiment was performed in duplicate, and then the recovery was calculated as the difference between the peak area of the standard solution and the nucleotide peak area in the solution after the desorption. Exemplary results for GmP are summarized in Table 3. The desorption varied widely, depending on both the solvent and the adsorbent used. 

GmP desorption was possible in each of the conditions tested and from each of the selected adsorbents but varied in terms of recovery and standard deviation (Table 3). The lowest values were obtained for water with high pH. This may be related to the need to use counter-ions in the elution solvent. In our case, these were acetate anions. Higher recoveries were obtained with low concentrations of ammonium acetate. In this case, however, the effect of the pH of the solvent on GmP elution is apparent. With increasing pH, the recovery rises, but the standard deviation also increases; consequently, the method’s repeatability decreases (Table 3). For these reasons, 10 mM CH_3_COONH_4_ at pH 10 and 10.5 were rejected.

**Figure 4:**
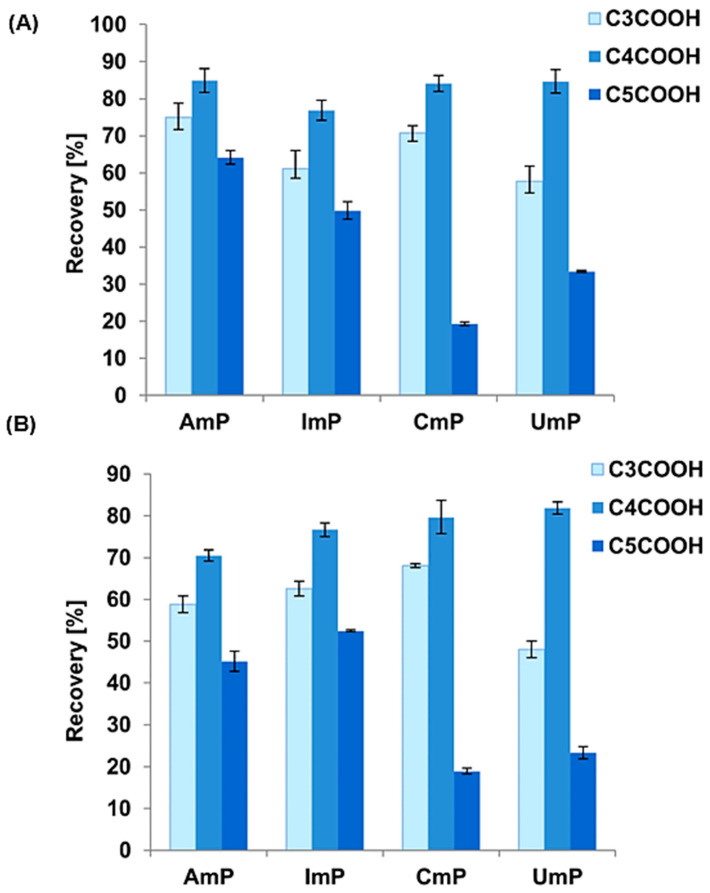
The recovery values for all the adsorbents used in the study and tested nucleotides desorbed with the use of 10 mM CH_3_COONH_4_ at pH 9 (**A**) and 9.5 (**B**) (UmP desorption from C3COOH was not tested).

Further testing for the remaining nucleotides was performed using 10 mM CH_3_COONH_4_ at pH 9 and 9.5. Figure 4 shows that the highest recoveries were obtained for 10 mM CH_3_COONH_4_ solution pH 9 and the C4COOH adsorbent (Figure 4).

There are evident differences in recovery depending on the adsorbent used. These differences are most significant if we compare the results for C3COOH and C5COOH with those for C4COOH (Figure 4). Apparently, it is from the surface of C4COOH that the greatest amount of nucleotides can be desorbed, and this adsorbent was chosen for the final extraction procedure. This result may be an effect of differences in the number of carbon atoms in the ligands of dicarboxylic acids bound to the surface of the material (it can be seen that in the case of an odd number, the recovery decreases, in contrast to an even number of carbon atoms) and also from differences in the efficiency of the synthesis. These differences may result from the mutual arrangement of two carboxylic groups (specifically, carbonyl and hydroxyl groups) in the even and odd carbon atom chains. Similar results were obtained by Stevenson et al. [23] regarding the adsorption phenomena of phenyl-bonded stationary phases with different even and odd alkyl chains. It is challenging to estimate unequivocally how many carboxyl groups are on the surface of the adsorbent, and it is the number of these groups that will largely determine the desorption of nucleotides. The lower the efficiency of the second stage of the synthesis, the fewer the carboxyl groups and the more residual amine groups there are. Not all nucleotides can be eluted from the surface of adsorbents due to the lower electrostatic repulsion part. For this study, C4COOH was finally chosen for the nucleotide extraction, and the pH of the acetate buffer was 9, because under these conditions, the recoveries for all nucleotides were at a similar level of about 80–85%. It should be noted that, for these conditions, the standard deviations did not exceed two percent, demonstrating the reproducibility of the method (Figure 4).

#### 3.3.4. The Final Optimized dSPE Method: Its Advantages, Limitations and Greenness

**Figure 5:**
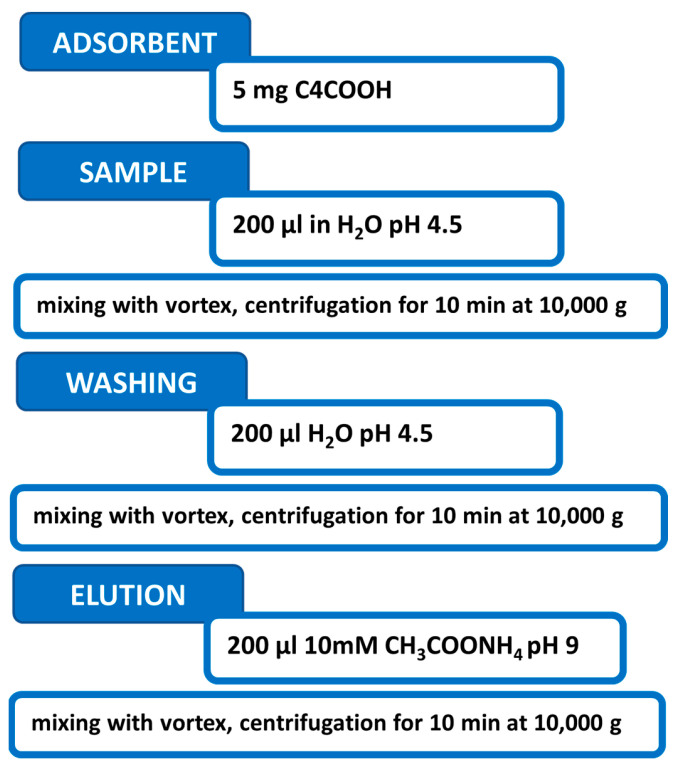
The method developed for nucleotide extraction with the use of the newly synthesized material.

The final optimized and applied dSPE extraction procedure is presented in Figure 5. The developed method has several significant advantages. First, it is simple, and the procedure is not time-consuming. There is no need to use inorganic salts or high concentrations of these salts, which often hinders the further analysis of the sample, e.g., when MS detection is used. The presence of inorganic salts makes it necessary to introduce an additional step to the sample preparation—desalting—which can cause further nucleotide loss.

It should be noted that the described method is based on the use of water and organic salts at low concentrations. Water is the most environmentally friendly solvent, but also the safest for the analyst. The developed method does not use any organic solvent, so we can call it a green and safe one. The method is based on using a weak ion exchanger, whose charge changes with the pH of the solution used for adsorption and desorption. Thus, it is possible to control the efficiency of the process by simply changing the pH. In our opinion, the cost of a single extraction will be lower since organic solvents are not used. At the same time, it should be emphasized that the recovery achieved by the developed dSPE method and its reproducibility are satisfactory.

We must also mention the disadvantages of the developed method. The critical parameter is the correct pH, and this must be strictly controlled, as any change will cause, for example, incomplete adsorption or reduced recovery. A complication may also be the need to centrifuge the solution three times, as it will be a challenge to scale up the process for larger sample sizes. However, the whole process can be carried out in one centrifuge tube. The limitation of the method is also the time needed for the extraction; without the proper centrifuge, a single centrifugation can take up to 30 min. The synthesized materials and the developed dSPE method are suitable for samples with a relatively high concentration of nucleotides. Its application to the concentration of these compounds is limited.

**Figure 6:**
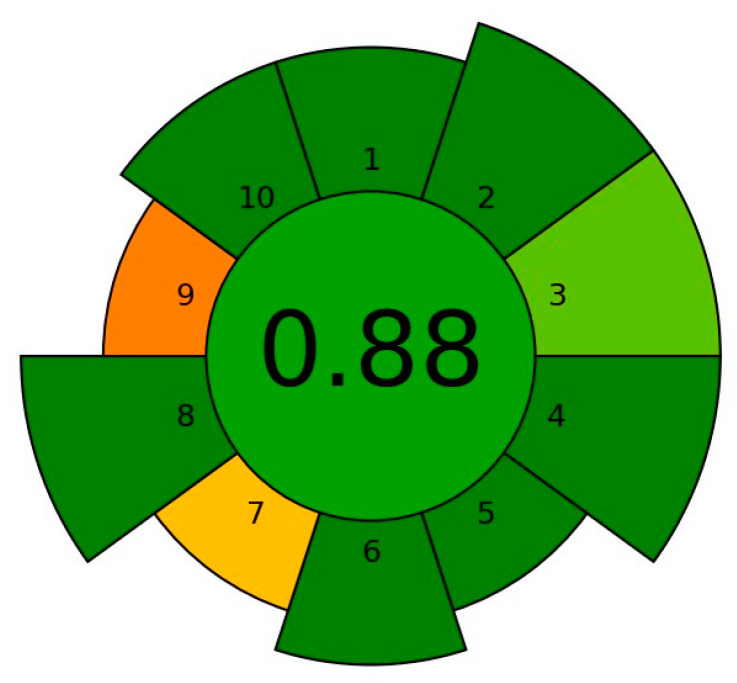
The result of the environmental assessment of the sample preparation method according to AGREE. Colors represent the scale of environmental friendliness from red (0) through yellow, orange to green (1).

The greenness of the method was determined according to the AGREEprep calculator (Gdańsk University of Technology, Gdańsk, Poland) [29], which is a part of AGREE software [30] dedicated to sample preparation methods. The calculator automatically adjusts the weights for each of the 10 principles to determine the relevance of the entered data for the segment. These principles are as follows: (1) sample preparation placement; (2) hazardous materials; (3) sustainability, renewability, and reusability of materials; (4) waste; (5) size economy of the sample; (6) sample throughput; (7) integration and automation; (8) energy consumption; (9) post-sample preparation configuration for analysis; (10) operator’s safety. The result for the environmental assessment of the developed method, according to the AGREE calculator, is listed in Figure 6. It contains the evaluation of each parameter individually, and the overall results of the procedure are placed in the center of the pictogram. Apparently, the application of liquid chromatography as a final determination method (principle 9) as well as the number of sample preparation steps and degree of automation (principle 7) lowered the greenness parameter. Ideally, the green method would have a value of 1; however, our sample preparation method obtained the high score of 0.88. This proves that the developed procedure fits into the principles of green analytical chemistry and may be also considered a safe method for the analyst. 

Table 4 collects basic information on the nucleotide extraction methods currently in use (types of solvents and adsorbents, recoveries, and matrices) and the method developed in this research. Compared with SPE and other dSPEs, the advantage of our method is the use of more environmentally friendly solvents (water, low-concentration organic salt). However, the time of the procedures is comparable (Table 4). Microwave-assisted extraction (MAE) is comparable to our method (use of water and organic acid), but only for a matrix such as an infant milk formula. If MAE is applied to a more complex matrix (lentil waste), then it is necessary to apply 80% methanol in water (Table 4). The application of dispersive magnetic solid-phase extraction (DMSPE) to the extraction of nucleotides provides better results compared with the method developed during the present study. Magnetic adsorbents shorten the time and simplify the extraction, while low-concentration salt or base solutions are used (Table 4).

### 3.4. Application of the Developed Extraction Method to Dietary Supplement Samples

**Figure 7:**
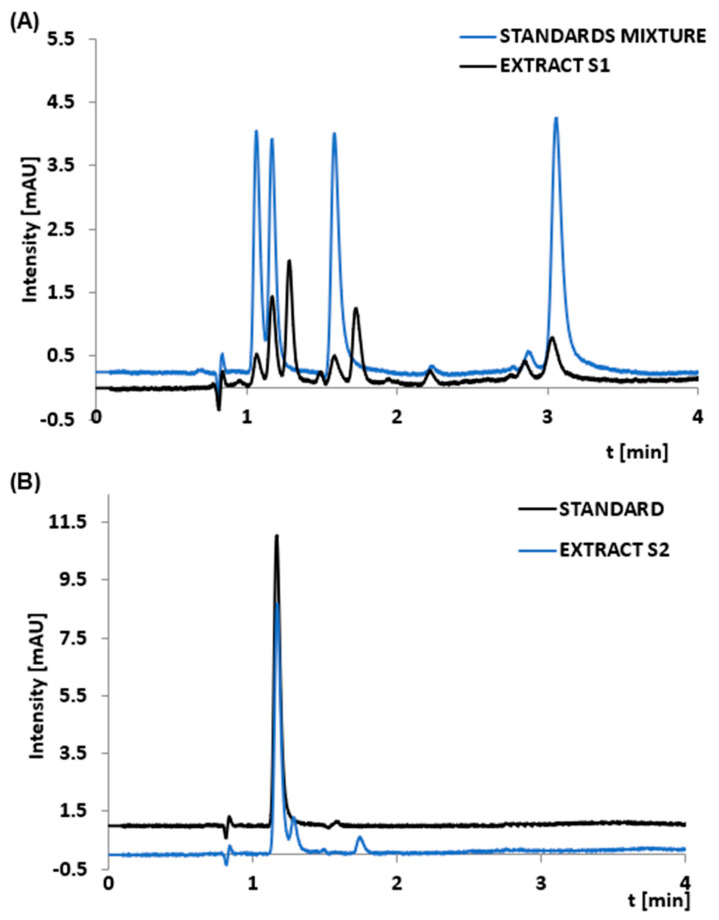
Chromatograms of the nucleotides standards and the dSPE extracts obtained from two different dietary supplements: S1 (**A**) and S2 (**B**).

The final stage of the study was to check whether the developed dSPE method, which gives promising results for nucleotide standards, also works when extracting these compounds from dietary supplement samples. Two different supplements from the local Polish market with various compositions were selected. These were selected based on differences in the matrix. S1 contained nucleotides (CmP, UmP, GmP, AmP) and insulin, while S2 contained UmP, three B vitamins, and folic acid. Exemplary chromatograms of extracts from S1 and S2 are shown in Figure 7. Two methods were used to identify nucleotides in the extracts. The first one is based on comparing retention times for the standards and extracts. When the result was not unambiguous, extracts were enriched with the standards.

As seen in the chromatogram from S1, the method developed during the study allowed for successful nucleotide extraction, and their quantitative determination was possible. However, complete sample purification was impossible (Figure 7A). The problem with the sample cleanup is probably due to the very complex composition of the tested samples (e.g., yeast strain). We suspect that some yeast cells may have adsorbed on the surface of the adsorbent. The use of a solvent at pH 9 for desorption may have led to lysis of these cells. Consequently, more compounds would have been present in the sample, resulting in a greater number of peaks on the chromatogram (these are compounds of medium polarity, such as other cellular nucleotides or nucleosides) (Figure 7A). However, our assumptions require additional, comprehensive, and complex studies, which will be undertaken in the next steps. Regarding the second supplement, UmP was successfully extracted from a preparation; however, other matrix-related compounds were also extracted (Figure 7B). These were vitamin B residues. The developed method allows for the extraction of nucleotides but does not provide high selectivity of extraction. In addition to nucleotides, other compounds from the supplements are also extracted. Apparently, unfortunately, not only anions (nucleotides) may adsorb on the surface. Other polar compounds present in the matrix adsorb on the sorbent surface, which may co-desorb from the material (Figure 7). Thus, it should be emphasized that the selectivity of the newly synthesized materials is not very high; nevertheless, it should be underlined that the developed extraction method makes it possible to extract nucleotides from dietary supplements of diverse compositions. Moreover, this method is very simple and certainly environmentally friendly.

The standard addition method was applied during the study to quantify the nucleotides in the supplements. Table S1 (Supplementary Materials) contains the equations of the calibration curves, the LOQ, linearity, and the working range of the calibration curve. Linearity was determined by calculating the determination coefficient (*R*^2^), which was higher than 0.996 (Table S1). The methods were characterized by high linearity within a certain range of concentrations and repeatability. The LOQ values were low.

The recovery of nucleotides from S1 and S2 was also determined. In the case of S2, it was 103 ± 2% for UmP. The recoveries of CmP, UmP, GmP, and AmP from the S1 supplement were as follows: 86 ± 1%, 102 ± 2%, 90 ± 3%, and 88 ± 1%, respectively. In the case of S1, the total content of nucleotides should equal 50 mg per capsule (information provided by the producer), but the results of our experiment proved that the content did not exceed 40 mg, with UmP being the highest. In the case of S2, the UmP content should be 50 mg per capsule, as indicated by the producer in the information provided. The content of this nucleotide was consistent with the declarations of their manufacturers.

In addition to the advantages of the developed method, which we have discussed in Section 3.3.4, it is also important to point out its advantages when it comes to the direct analysis of dietary supplements. Nucleotide extraction from nucleotide-containing samples (like one of the supplements we tested) usually involves several steps, such as homogenization with PBS, the application of 5% perchloric acid, 3 M KOH, several consecutive sample centrifugation steps, and filtration [36]. Sample preparation is therefore a multi-step process and requires the use of reagents that are both harmful to humans and the environment, unlike the method developed during the present study. Moreover, sample purification is a significant limitation of that method [36]. In the case of dietary energy supplements containing only one nucleotide and no other additives, sample preparation is very simple, because the tablet can be ground, dissolved in water, and filtered [37]. Admittedly, that method is simpler than the one we developed, but it is limited in its applicability to supplements containing only one group of compounds—nucleotides [37]. There are very few such supplements today, because the composition of most of them is more complex and contains compounds belonging to different groups (e.g., nucleotides, vitamins, yeast, organic acids). Thus, despite its simplicity, that method could not be applied to the supplements described in this publication, while the method we have developed, works well for supplements of complex compositions. With regard to food supplements, sample preparation usually involves dissolution in saline buffer and ultrasound extraction (15 min–1 h). Next, centrifugal filtration is performed using Amicon Ultra filters with a 10 kDa cut-off [38]. The significant limitation here is the lack of any sample purification, since all molecules up to 10kDa are present in the extract, not just nucleotides [38]. With the method we have developed, the sample purity is greater, the time is shorter, and the solvents used are equally environmentally friendly.

## 4. Conclusions

As there is still a need to improve sample preparation methods and search for new materials for extraction, our research is in line with both these trends. Another trend observed in analytical chemistry is the development of methods that meet the criteria of green chemistry, so that extraction without organic solvents can be possible. Green chemistry rules were essential to us during the study and in our attempt to synthesize new materials for the extraction of nucleotides. These materials possess amino- and carboxyl-terminal groups at the surface. We have shown, for the first time, that they can successfully isolate the studied compounds. At the same time, we found that the recovery of nucleotides depends on the number of carbon atoms in the ligand-bound structure in the second synthesis stage (the recovery was 10 to 50% greater for C4COOH than for C5COOH and C3COOH). The highest extraction efficiency was obtained for dicarboxylic acid with four carbons.

On the other hand, the binding of these acids enabled us to perform extractions involving adsorption and desorption based on electrostatic attraction or repulsion by changing the pH of the solutions used in the process. Therefore, water acidified to pH 4.5 with acetic acid was used during the adsorption of nucleotides because of the protonation of amino groups and the electrostatic attraction of nucleotides. The basic solution of ammonium acetate (pH 9) was used for the desorption due to the ionization of carboxyl groups and the electrostatic repulsion of nucleotide anions. Consequently, the extraction principle is very simple: it uses a low-concentration organic salt, and an acidic or alkaline pH is sufficient to extract nucleotides with high recovery and reproducibility (78–86%). Moreover, the application of dSPE makes it possible to carry out the whole process in one centrifuge vial. The developed method does not require the application of any organic solvent; therefore, it may be considered environmentally friendly and cheaper than conventional methods (LLE or SPE). This extraction procedure is novel in the study of nucleotides. 

The developed extraction method may be successfully used for dietary supplements differing in composition. We have shown that nucleotide extraction was possible even in the case of a supplement whose composition included yeast strains. The material and procedure did not allow for the complete purification of the sample, but it was nevertheless possible to extract and quantitatively and qualitatively analyze the nucleotides in the supplement. The method has many advantages (e.g., simplicity, short time, without organic solvents, the whole process in one vial, high recoveries, and reproducibility). It can be successfully applied to real samples now and in the future. 

## Figures and Tables

**Table 1:** Results of elementary analyses: the content of carbon, nitrogen, and hydrogen, and the calculated coverage density of bonded groups.

	Adsorbent			
	Di-Amine	C3COOH	C4COOH	C5COOH
Carbon content [%]	6.267	7.300	8.184	9.164
Hydrogen content [%]	2.709	2.018	2.506	2.103
Nitrogen content [%]	2.547	2.196	2.112	2.115
Coverage density [µmol/m^2^]	2.56	0.59	0.83	1.02

**Table 2:** Summary of the results of the study of nucleotide adsorption on the surface of synthesized materials.

Solvent	Adsorbent	Nucleotide	Percentage of Nucleotide Adsorbed at the Surface [%]
H_2_O pH = 4.5 (adjusted with acetic acid)	C3COOH	GmP	97.4 ± 0.4
		AmP	81.7 ± 2.9
	C4COOH	GmP	96.4 ± 0.4
		AmP	99.8 ± 1.6
	C5COOH	GmP	90.1 ± 1.8
		AmP	81.1 ± 4.8
10 mM CH_3_COONH_4_ pH = 4.5 (adjusted with acetic acid)	C3COOH	GmP	85.5 ± 5.6
		AmP	92.6 ± 0.6
	C4COOH	GmP	90.5 ± 0.2
		AmP	82.5 ± 0.4
	C5COOH	GmP	92.1 ± 4.8
		AmP	95.5 ± 5.6
10 mM CH_3_COONH_4_ pH = 5.5 (adjusted with acetic acid)	C3COOH	GmP	80.8 ± 0.2
		AmP	77.1 ± 1.0
	C4COOH	GmP	88.8 ± 0.4
		AmP	86.9 ± 0.6
	C5COOH	GmP	80.0 ± 4.8
		AmP	75.5 ± 5.6

**Table 3:** Summary of exemplary desorption results for GmP.

Solvent	Adsorbent	Percentage of Desorbed Nucleotide [%]
10 mM CH_3_COONH_4_ pH = 9	C3COOH	67.5 ± 0.6
	C4COOH	79.8 ± 1.9
	C5COOH	67.9 ± 3.3
10 mM CH_3_COONH_4_ pH = 9.5	C3COOH	67.3 ± 2.8
	C4COOH	72.8 ± 1.7
	C5COOH	67.1 ± 2.9
10 mM CH_3_COONH_4_ pH = 10	C3COOH	75.3 ± 12.3
	C4COOH	85.0 ± 9.9
	C5COOH	73.0 ± 6.4
10 mM CH_3_COONH_4_ pH = 10.5	C3COOH	70.3 ± 4.7
	C4COOH	73.2 ± 5.3
	C5COOH	85.5 ± 6.5
H_2_O pH = 10	C3COOH	19.8 ± 1.7
	C4COOH	65.4 ± 1.7
	C5COOH	73.5 ± 0.6
H_2_O pH = 10.5	C3COOH	4.0 ± 0.2
	C4COOH	15.2 ± 0.5
	C5COOH	44.2 ± 1.3

**Table 4:** Comparison of methods used for nucleotide extraction with the method developed in the present study.

Method	Adsorbent	Solvents	Recovery [%]	Time	Matrix	Reference
DMSPE	magnetic ferroferric oxide nanoparticles, Fe_3_O_4_@GO	0.05 M NaOH pH 3.5	88–109	~20 min	plant	[31]
DMSPE	titanium-ion-functionalized ferroferric oxide magnetic particles	0.02 M Na_3_PO_4_·12H_2_O	76–95	~20 min	plant	[32]
dSPE	activated charcoal	ethanol, water, 1 M HCl, 2% NH_3_, and 50% acetonitrile in water	—	~1 h	bacteria	[22]
SPE	Strata X	0.025 M ethanolamine pH 8.0, water, methanol	86–98	~1 h	cerebrospinal fluid	[18]
MAE	—	MeOH:H_2_O 80:20 (*v*/*v*)	—	~1 h	lentil waste	[33]
MAE	not used	water, formic acid	95–104	~30 min	infant formulas	[34]
SPE	strong cation exchanger	0.5 M KH_2_PO_4_ pH 3.0; 0.3 M KBr	81–102	~30 min	infant formulas	[35]
SPE	anion-exchanging	0.1–0.3 M NH_4_H_2_PO_4_ (pH 3.0), NH_4_OH (pH 10.0)	—	~1 h	phospho-peptides	[14]
SPE	polymeric reversed-phase Strata-X	25 mM ethanolamine (pH 5); 30% methanol in 25 mM ethanolamine (pH 5)	98–104		renal cell line and urine	[19]
dSPE	weak anion exchanger	water (pH 4.5), 0.01 M CH3COONH4 (pH 9.0)	78–87	~40 min	dietary supplements	our method

“—” Not used.

## Data Availability

The data used to support the findings of this study can be made available by the corresponding author upon request.

## Supplementary Materials

The following supporting information can be downloaded at: https://www.mdpi.com/article/10.3390/foods12193675/s1, Figure S1. FT IR spectra of Di-amine (a), C3COOH (b), C4COOH (c), and C5COOH (d); Figure S2. 13C NMR spectra of Di-amine (A), C3COOH (B), C4COOH (C), and C5COOH (D); Figure S3. Chromatograms for nucleotides and their mixtures: (A) CmP, UmP, GmP, ImP and AmP standards; mobile phase: 50 mM CH3COONH4 (pH = 5.5) and methanol gradient elution: 0–10 min 0–10% MeOH; (B) separation of a mixture of nucleotide standards: CmP, UmP, GmP and AmP;mobile phase: 50 mM CH3COONH4 (pH = 5.0) and methanol gradient elution 0–5 min 0–5% v/v MeOH. Other experimental conditions: C18PFP column, column temperature 30 °C, mobile phase flow rate 0.3 mL/min, injection volume 1 μL, UV detection, wavelength 254 nm; Figure S4: Kinetics of AmP and GmP adsorption at C4COOH surface, Table S1. Calibration curves equations for two different dietary supplements (S1 and S2) determined with the use of the standard addition method. Each injection was repeated three times.
