# Supplementary material for: Extraction of Nucleotides from Dietary Supplements by Newly Synthesized Adsorbents
Source: Foods. 2023 Oct 6;12(19):3675. doi: 10.3390/foods12193675 (PMC10572691; doi:10.3390/foods12193675)
Supplement: Supplementary file 1 [file foods-12-03675-s001.zip › foods-2572307-supplementary.pdf]

Synthesis of new adsorbents and their application to the extraction of nucleotides from dietary supplements

Sylwia Studzińska[a,*], Paulina Stypczyńska[a], Szymon Bocian[a], Andrzej Wolan[b]

[a]Chair of Environmental Chemistry and Bioanalytics, Faculty of Chemistry,
Nicolaus Copernicus University in Toruń,
7 Gagarin Str., PL-87-100 Toruń (Poland),
tel. (48)(56)6114308, fax. (48)(56)6114837,
[b]Chair of Organic Chemistry, Faculty of Chemistry,
Nicolaus Copernicus University in Toruń,
7 Gagarin Str., PL-87-100 Toruń (Poland)

*e-mail: kowalska@chem.umk.pl

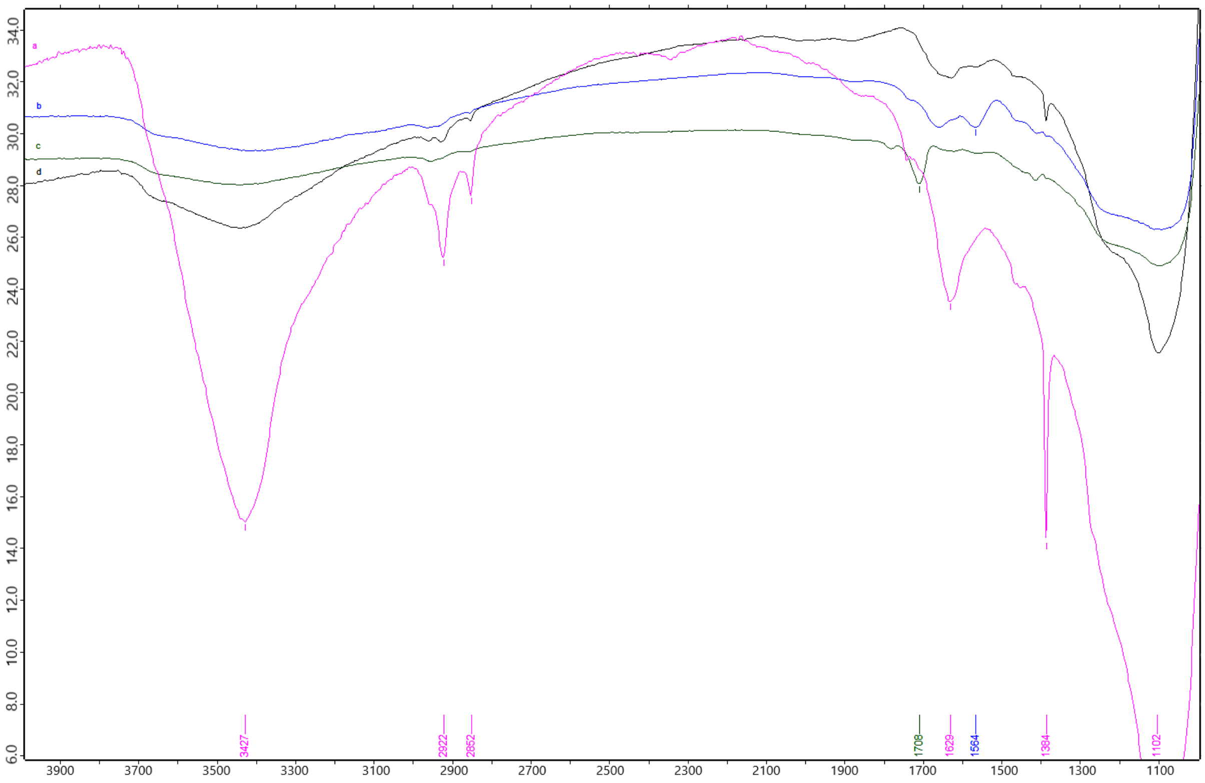

Figure S1. FT IR spectra of Di-amine (a), C3COOH (b), C4COOH (c), and C5COOH (d).

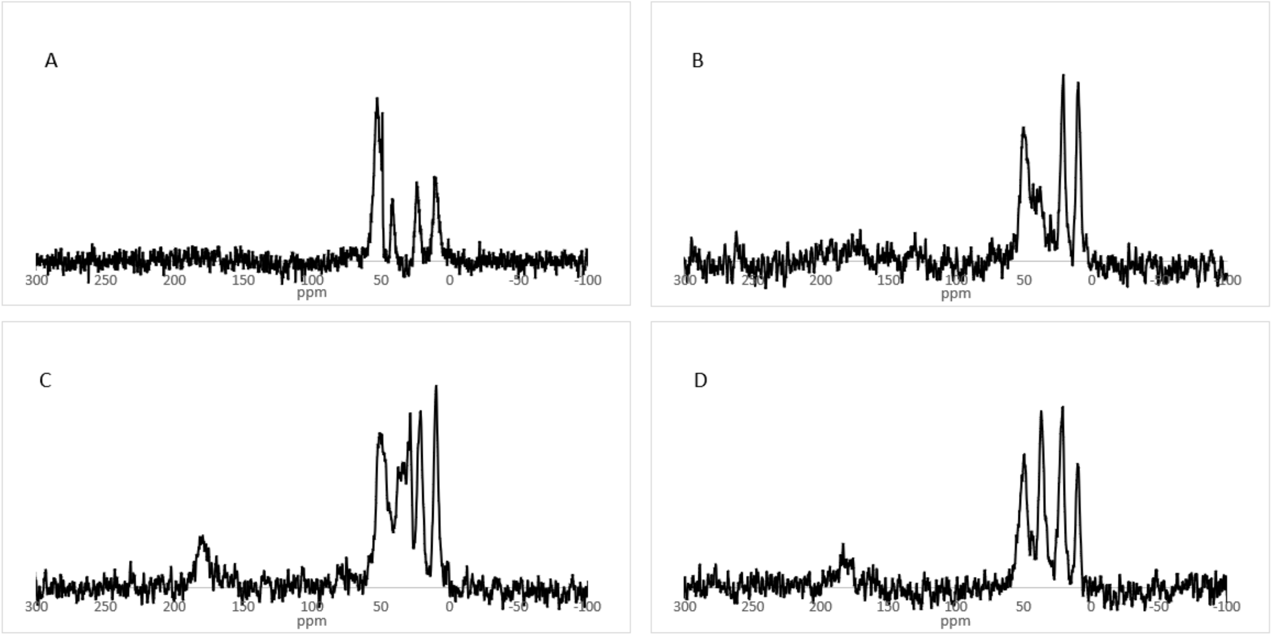

Figure S2. 13C NMR spectra of Di-amine (A), C3COOH (B), C4COOH (C), and C5COOH (D).

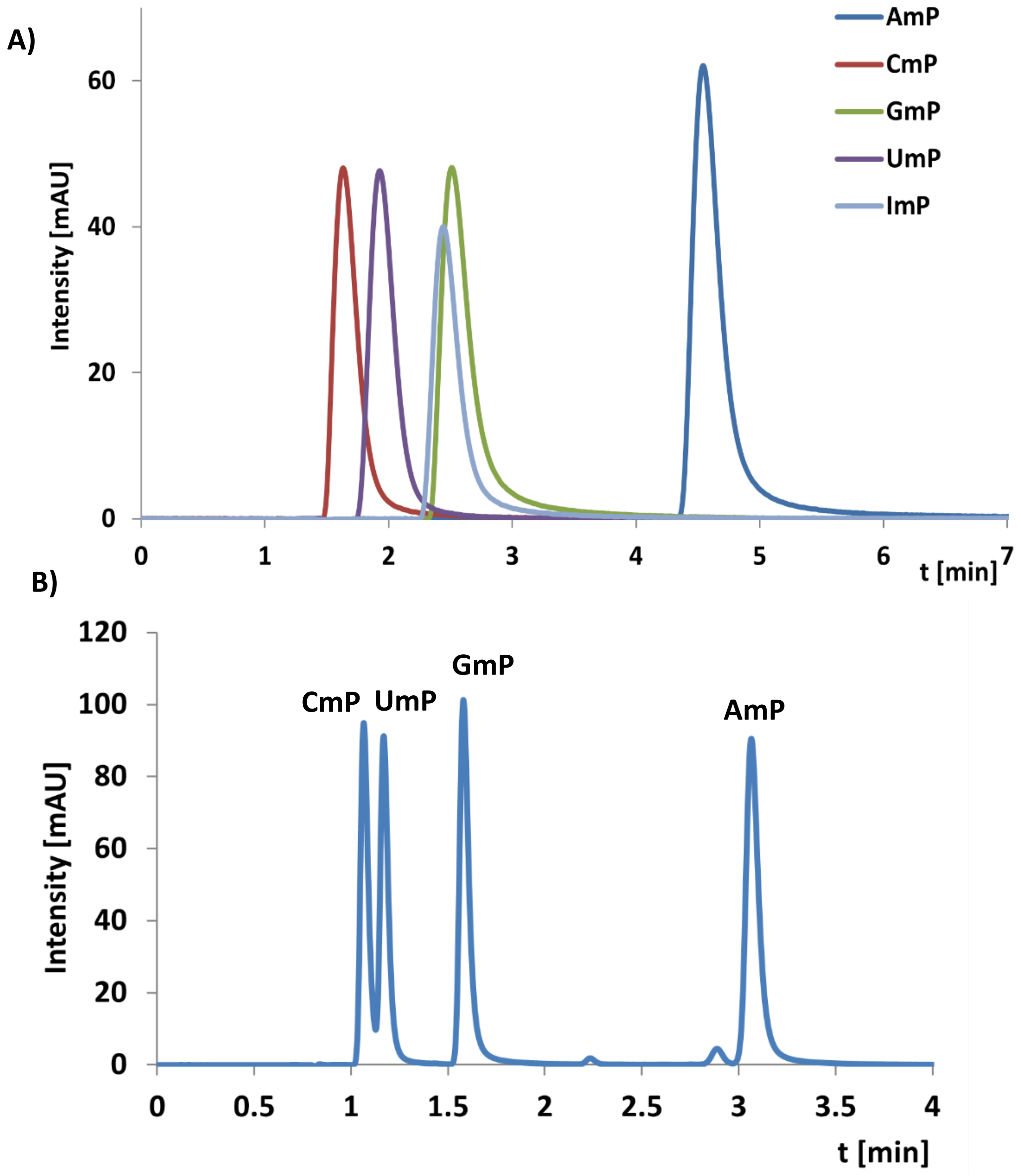

Figure S3. Chromatograms for nucleotides and their mixtures: A) CmP, UmP, GmP, ImP and AmP standards, mobile phase: 50mM CH_3COONH_4 (pH=5.5) and methanol, gradient elution: 0-10 minutes 0-10% MeOH; B) separation of a mixture of nucleotide standards CmP, UmP, GmP and AmP, mobile phase: 50mM CH_3COONH_4 (pH=5.0) and methanol, gradient elution 0-5 minutes 0-5% v/v MeOH. Other experimental conditions: C18PFP column, column temperature 30°C, mobile phase flow rate 0.3 ml/min, injection volume 1 µl, UV detection, wavelength 254 nm.

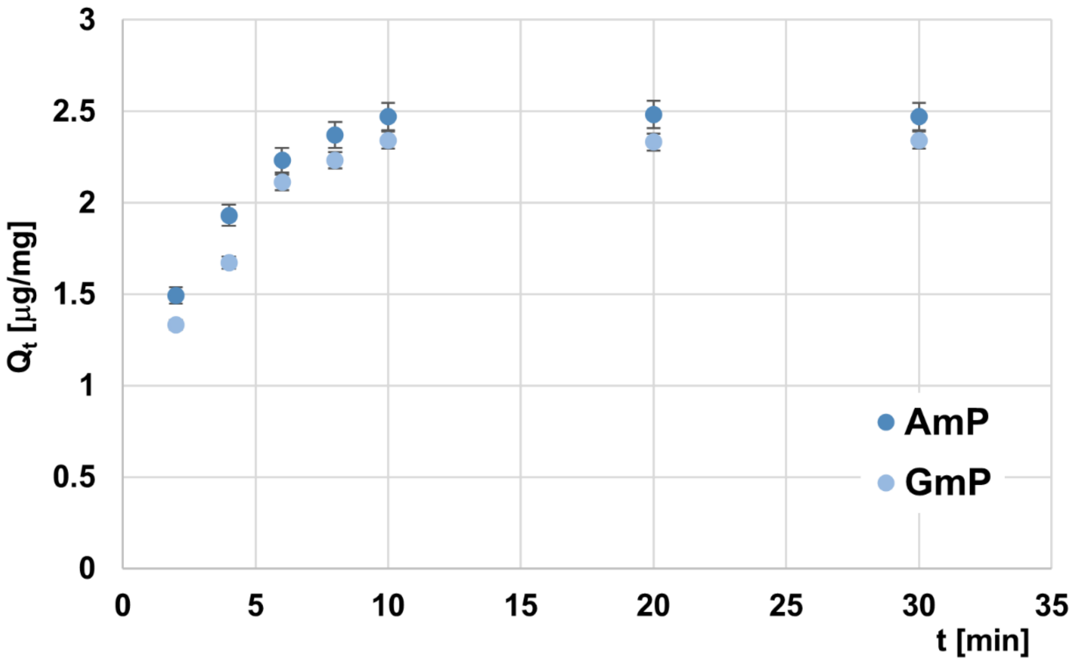

Figure S4. Kinetics of AmP and GmP adsorption at C4COOH surface.

Table S1. Calibration curves equations for two different dietary supplements (S1 and S2) determined with the use of the standard addition method. Each injection was repeated three times.

Analyte	LOQ [µg/ml]	S1			S2		
		Calibration curve equation	R^2	Concentration range [µg/ml]	Calibration curve equation	R^2	Concentration range [µg/ml]
CMP	0.25±0.06	y=891.7 (±2.4)x+532.3 (±0.9)	0.997	2.5-20.0	–	–	–
UMP	0.33±0.05	y=1003.0 (±5.9)x+2044.8 (±7.1)	0.998	7.5-40.0	y=1211.00(±21.2)x+ 1061.4 (±12.4)	0.997	2.5-25
GMP	0.20±0.03	y=1238.4 (±2.7)x+897.2 (±3.4)	0.998	2.5-20	–	–	–
AMP	0.40±0.05	y=1467.8 (±5.3)x+1381.2 (1.1)	0.999	5.0-45.0	–	–	–
